# On the Treatment of Aural Suppuration. II.—The Chronic Form

**Published:** 1893-10-21

**Authors:** L. H. Pegler


### The Hospital Clinic.

[*The Editor will be glad to receive offers of co-operation and contributions from members of the profession. All letters should be addressed to* The Editor, The Lodge, Porchester Square, London, W.]

### ST. MARY'S HOSPITAL.

### The Treatment of Aural Suppuration.

### II.—The Chronic Form.

Before passing to the consideration of chronic otorrhœa, it is advisable to remind the reader, in order to avoid any misapprehension, that a chronic discharge from the external auditory meatus is not necessarily the sequel of some previous attack of acute median otitis that has resisted treatment, or been suffered to continue unchecked. The suppuration may have originated from inflammation, primarily involving (1) the external meatus; (2) the tympanic membrane. Primary inflammation of the external meatus is known as *otitis externa*, and it appears in a variety of forms, including a circumscribed and a diffuse, the latter of which may be acute or chronic. Circumscribed otitis is better known as boil in the meatus, or *furuncle*, and it is when there are several of these little boils in close approximation to each other that the *diffuse* form is said to occur, constituting the diffuse *furunculosis* of authors. This is a common disease in our out-patient rooms; the soft structures lining the meatus are infiltrated and tumefied, and a free secretion of pus occludes its lumen. Under these circumstances, it is not surprising that if this state of things is not checked it may drift into a chronic condition, and implicate the tympanic cavity secondarily by perforation through the membrane. By scrupulous attention to cleanliness, and the free application of a strong solution of boric acid in spirit of wine to the diseased surfaces, we endeavour to arrest the morbid processes and preserve that important organ intact. (2) Primary *myringitis*, and inflammation of the membrana tympani is designated may be the result of a traumatism—of mould—*aspergillus*, *mucor*—in the meatus, exposure to cold, or the entrance of cold water into the ear, as is apt to occur in bathing. If the inflammatory action is sufficiently intense, perforation of the membrane may supervene, the middle ear becomes involved, and a sort of vicious circle is set up, resulting in chronic suppuration. Thus it will readily be believed that a case of the latter coming before us, it may be next to impossible to state from outward observation whether the external or middle ear was the original seat of attack.

Having now arrived at a tolerably clear understanding as regards the etiology of an otorrhœa, we are in a better position to proceed with the consideration of chronic suppuration of the middle ear. We are dealing now with a perforation of the drum membrane that has thus far refused to close. We may safely start with that assumption, whether occurring in a child, or in an adult, and from whatever cause arising, in other words, whether a sequent of an acute attack of median otitis, or commencing insidiously by extension from disease situated more externally, a perforation, large or small, is at the bottom of the mischief. After carefully mopping away discharge with a cotton holder, the aperture may generally be seen without difficulty by the aid of a speculum, and if the patient is directed to "hold his nose and blow," some thin secretion will generally be seen to bubble through it (unless the Eustachian tube is not sufficiently patent), often making a squeaking noise in exit. This is as it should be, and shows there is no hindrance to its escape. The appearance presented now is quite different from what was described as occurring in an acute case. The redness and congestion of the membrane, rendered still more conspicuous by the partial adherence of a whitish epithelial débris, has now disappeared, and unless almost entirely destroyed by the ulcerative process, more or less healthy-looking membrane (except in extreme cases to be presently described) may usually be observed, with the malleus in situ. It rarely happens that a recent perforation refuses to close up, but once this chronic condition has been suffered to establish itself, a happy termination is by no means so frequent. The discharge has lost now its glairy mucopurulent character, and has become thinner in consistence, more irritating, and usually extremely malodorous; this alteration is probably owing to certain specific micro-organisms—streptococci, staphylococci—having gained access to the suppurating surfaces from without. Unfortunately, if closure of the perforation does take place at this late stage, it is at the expense of the integrity of the hearing power to a greater or less extent, and permanent cicatrices remain behind, marking its original site. When the hole has been a large one, adhesions of the remnant to the promontory are often formed, the handle of the malleus permitting the approximation of the two planes, owing to its retraction inwards by the tensor tympani muscle. In certain cases, especially those following a severe attack of scarlet fever or diphtheria, the perforation is too extensive to cicatrise, a mere rim of the membrane surviving, in which case the exposed mucous surface of the inner wall of the tympanum may become dry and sclerosed. In fact, the variety of appearances one meets with is infinite, but the hearing is always more or less seriously impaired. In many subjects there appears to be a peculiar predisposition to the growth of exuberant granulations, and then the secretions are apt to be more or less tinged with blood. If this exuberant granulation tissue is suffered to over develop, it may become polypoid, and may eventually grow into a true polypus; this is probably the origin of most aural polypi, their precise situation varying according to circumstances. It only remains to mention a class of case in which perforation has taken place through the upper segment of the drumhead, called Schrapnell's membrane, or *membrana flaccida*. The hole is generally small, is often covered over by a crust of dried blood or pus, and the discharge through it is not very profuse, but immediately behind the aperture is the head of the malleus, and it invariably feels rough when touched with a probe, indicating a carious implication of its surface.

The treatment of acute and chronic suppuration is based upon precisely similar principles, the difference is mainly one of detail. It is usual at St. Mary's, after thoroughly removing every trace of purulent secretion, to prescribe a lotion for instillation; Mr. Field usually commences with the following, which combines a disinfectant and deodoriser with a mild astringent: ℞. Zinci sulphatis gr. v., acid carbolic gr. v., aquam ad ℥j. Sig. To be instilled warmed into the ear, after syringing and drying. In certain cases, or as alternatives, the following are frequently employed, ℞. Acid boric gr. v.—xx., aq. dest. ad. ℥j; or ℞ Acid carbolic, gr. v.—x., aq. dest. ad. ℥j. It is seldom advisable to continue the use of the same lotion for more than one fortnight at a time, and one secret of success consists in a knowledge of how to succeed one agent by another until a cure is effected. If the foregoing have failed, a trial may be made of the biniodide of mercury lotion, strength, one to a thousand. This drug is best employed in the form known as iodic hydrarg, a soluble preparation of the biniodide, made by Messrs. Burroughs and Wellcome at the suggestion of Dr. Luff, of St. Mary's Hospital. It has an advantage over the perchloride in not precipitating the albuminous constituents of the secretions. Mercurial applications must necessarily be ordered of somewhat feeble strength, for it must be remembered that a certain proportion of the drops is apt to find its way into the pharynx through the Eustachian tube, if they have been instilled into the meatus in such a way as to be of service remedially. It is customary at St. Mary's to direct the patient to rest his head, with the affected side up, on some suitable support; then the warm lotion being poured into the ear by an attendant from a spoon, or squeezed by himself out of a piece of saturated cotton wool, he performs the Valsalvan experiment (*i.e.*, compresses his nose with the fingers and thumb of his disengaged hand, and blows out his cheeks); a rarefaction of the air in the cavity of the tympanum is created by virtue of its escape through the perforation and the fluid in the meatus, when the latter, rushing in by suction, is brought into direct contact with the affected parts. The biniodide is an especial favourite with Dr. William Hill, who also frequently prescribes the sulphate of soda as a lotion (gr. x—xx. in ℥j. aq.). When granulations exist nothing is more serviceable than rectified spirit associated with boric or salicylic acid, and less and less diluted until it can be borne at B.P. strength.

The following is a good combination: ℞. Pulv. acid boric, ʒi.; spt. vin. rect., ʒj., aq. destil, ad. ℥j. This does not, of course, form a complete solution, and must be shaken up well before use. It may be followed in a week or so, if unsuccessful, by a stronger formula, of Mr. G. P. Field, as thus: ℞. Acid salycilic, gr. x.; acid boric, gr. xx.; glycerini, ʒij.; spt. vini. rect. ad. ℥j. Under treatment by one or other or all of the preceding, alternated with each other if necessary, a large number of otorrhœas are cured. It will, of course, be understood that cases coming under observation, in which exuberant or polypoid granulations are keeping up the suppuration, the former, if too large to be shrunk by the spirit within a reasonable period, are first curetted, or touched with nitrate of silver or chromic acid, preference being given to the last-named. Iodoform, so highly regarded still as an antiseptic, &c., for surgical dressings, has had its trial at St. Mary's in aural practice, but has been discarded for intrameatal use, owing to its failure in checking the growth of granulations.

L. H. Pegler, M.D.

